# A phase I study of ABT-510 plus bevacizumab in advanced solid tumors

**DOI:** 10.1002/cam4.65

**Published:** 2013-03-21

**Authors:** Hope E Uronis, Stephanie M Cushman, Johanna C Bendell, Gerard C Blobe, Michael A Morse, Andrew B Nixon, Andrew Dellinger, Mark D Starr, Haiyan Li, Kellen Meadows, Jon Gockerman, Herbert Pang, Herbert I Hurwitz

**Affiliations:** 1 Duke University Medical CenterDurham, North Carolina, 27710; 2 Sarah Cannon Research InstituteNashville, Tennessee

**Keywords:** ABT-510, advanced solid tumors, bevacizumab, phase I

## Abstract

Targeting multiple regulators of tumor angiogenesis have the potential to improve treatment efficacy. Bevacizumab is a monoclonal antibody directed against vascular endothelial growth factor and ABT-510 is a synthetic analog of thrombospondin, an endogenous angiogenesis inhibitor. Dual inhibition may result in additional benefit. We evaluated the safety, tolerability, and efficacy of the combination of bevacizumab plus ABT-510 in patients with refractory solid tumors. We also explored the effects of these agents on plasma-based biomarkers and wound angiogenesis. Thirty-four evaluable subjects were enrolled and received study drug. Therapy was well tolerated; minimal treatment-related grade 3/4 toxicity was observed. One patient treated at dose level 1 had a partial response and five other patients treated at the recommended phase II dose had prolonged stable disease for more than 1 year. Biomarker evaluation revealed increased levels of D-dimer, von Willebrand factor, placental growth factor, and stromal-derived factor 1 in response to treatment with the combination of bevacizumab and ABT-510. Data suggest that continued evaluation of combination antiangiogenesis therapies may be clinically useful.

## Introduction

Angiogenesis, the process of new blood vessel formation, plays a critical role in the growth of tumors. The process by which tumors develop this angiogenic phenotype has been termed the “angiogenic switch” [1]. Numerous antiangiogenesis strategies have been developed, including the targeting of proangiogenesis factors such as vascular endothelial growth factor (VEGF) or its receptors. Naturally occurring proteins and peptides that possess antiangiogenic properties, including thrombospondin (TSP) and its analogs, are also being studied as cancer therapies. The impact of combining agents with complementary antiangiogenesis mechanisms of action has not been well studied in patients.

Bevacizumab is a recombinant, humanized, monoclonal antibody directed against VEGF [2]. VEGF is known to play a pivotal role in tumor angiogenesis and is a significant mitogenic stimulus for arterial, venous, and lymphatic endothelial cells (ECs). VEGF can induce vascular permeability essential for extracellular remodeling and can serve as an EC survival factor. Bevacizumab is approved for use in the treatment of several tumor types [3].

TSP-1 was the first recognized endogenous inhibitor of angiogenesis [4] and has been shown to inhibit neovascularization and tumorigenesis in numerous preclinical models [5]. The mechanisms underlying the antiangiogenic effects of TSP are not well understood but altered signaling via various integrins, CD36, and CD47 have been implicated as well as sequestration of proangiogenic factors such as VEGF and basic fibroblast growth factor [6, 7]. Most of the antiangiogenic activity of TSP has been localized to short amino acid sequences known as TSP repeats [7]. ABT-510 is a synthetic analog of an N-terminal region of TSP-1, with amino acid substitutions that provide greater stability and enhance the antiangiogenic properties of native TSP-1 [8]. In vitro, ABT-510 inhibits chemotactic VEGF–stimulated migration of human microvascular ECs, EC proliferation, and tube formation. ABT-510 also induces EC apoptosis at nanomolar concentrations [5].

ABT-510 has been studied both as a single agent and in combination with cytotoxic chemotherapies, including 5-fluorouracil/leucovorin and gemcitabine/cisplatin [9–11]. However, ABT-510 has not been studied in combination with other targeted agents. We sought to evaluate the impact of combining ABT-510 plus bevacizumab, thereby targeting both sides of the “angiogenic switch” to achieve more complete inhibition of angiogenesis and, potentially, greater control of tumor growth. The objectives of this phase I study were to evaluate dose limiting and non-dose limiting toxicities of this combination and to determine the recommended phase II dose (RPTD) for the combination of ABT-510 plus bevacizumab in patients with advanced solid tumors. The study consisted of two phases, a dose escalation phase meant to identify a RPTD and a biomarker expanded cohort at the RPTD, which was meant to better describe the effect of this regimen on several markers of angiogenesis. During the biomarker expanded cohort, patients received either monotherapy ABT-510 or monotherapy bevacizumab followed by the combination of both agents. The pharmacodynamic effects of drug treatment(s) were evaluated via two methods: multiplex enzyme-linked immunosorbent assay (ELISA) measurement of blood-based angiogenic factors and a dermal wound angiogenesis assay.

## Patients and Methods

### Patient selection

Patients with histologically or cytologically confirmed solid tumor malignancies refractory to standard therapy or for whom there is no standard therapy were eligible for enrollment. Additional inclusion criteria included: age ≥18 years; Karnofsky performance status ≥70%; normal organ and marrow function defined by the following: absolute neutrophil count (ANC) ≥1500/mm^3^, platelets ≥100 000/mm^3^, hemoglobin ≥9.0 g/dL; aspartate aminotransferase (AST), alanine aminotransferase (ALT), ≤2.5 × upper normal limit (ULN) or ≤5 × ULN if known hepatic metastasis; bilirubin ≤1.5 × UNL; urine protein: creatinine ratio (UPCR) ≤1.0, creatinine clearance estimated ≥50 mL/min/1.73 m^2^; prothrombin time, international normalized ratio and partial thromboplastin time (PT/INR/PTT) ≤1.2 × ULN.

Patients were excluded if they received prior chemotherapy, radiation therapy, hormonal, biological therapy, or investigational agents within 28 days prior to day 1 of study drug or if they had major surgery within 28 days or minor surgical procedures within 14 days prior to day 1 of study drug. Additional exclusion criteria included: known brain metastases; poorly controlled or clinically significant atherosclerotic vascular disease, intraabdominal fistula or abscess, gastrointestinal perforation, or bleeding event (either gastrointestinal or pulmonary) >1 tablespoon within 6 months of day 1 of study drug; squamous cell carcinoma of the lung; history of intolerance of prior treatment with ABT-510 or bevacizumab; history of thrombosis within 3 months of day 1 of study drug or current therapeutic anticoagulation; poorly controlled hypertension (>160/100); significant previous treatment-related toxicities that were unresolved; and serious or poorly controlled medical conditions which could be exacerbated or that would seriously complicate compliance with the protocol. Women of child-bearing potential were required to practice contraception and could not be pregnant or lactating at any time during the study. This study was approved by the Duke Institutional Review Board, followed the guidelines of the Helsinki agreement, and was registered with http://ClinicalTrials.gov (NCT00888043). All patients provided informed written consent for study participation.

### Drug administration

Bevacizumab (Avastin®, Genentech/Hoffman La-Roche, Basel, Switzerland) was administered as a bi-weekly intravenous (IV) infusion on days 1 and 15 of each 28-day cycle. The initial infusion was delivered over 90 minutes with subsequent infusions given over 60 minutes and then 30 minutes if well tolerated. ABT-510 (Abbott Laboratories, Chicago, IL) was administered by twice daily subcutaneous (SC) injections; patients were trained to administer the SC injections at home.

Each new cycle could begin only if the subject met initial inclusion requirements for hematologic, hepatic, and renal assessments. There were no dose modifications for either drug; both drugs were to be held for toxicity occurrence unless the toxicity could be attributed primarily to only one agent (e.g., hypertension for bevacizumab). Modifications were to be made based on the most severe toxicity.

### Dose escalation

The dose escalation scheme is outlined in Table 1. Dose-limiting toxicities (DLTs) were evaluated during cycle one and were defined as follows: hematological toxicity ≥grade 4 neutropenia or thrombocytopenia; grade 3 hypertension that was not adequately controlled with the addition of up to 2 antihypertension medications and grade 4 hypertension; nausea/vomiting or diarrhea ≥grade 3 lasting >4 days despite adequate supportive care; grade 4 proteinuria or persistent grade 3 proteinuria >2.0 after discontinuation of bevacizumab; other clinically significant nonhematological toxicity ≥grade 3; any treatment-related death or hospitalization.

**Table 1:** Dose escalation scheme and cycle 1 dose limiting toxicity events (DLTs)

Cohort level	ABT-510 (mg, twice daily)	Bevacizumab (mg/kg, every 14 days)	No. of patients	No. of DLTs	DLT events
1	50	5	3	0	
2	50	10	3	0	
3	100	10	6	1	Grade 2 GI bleed
Expanded cohort	100	10	22	0	

GI, gastrointestinal.

Dose escalation used the standard “3 + 3” design up to full dose of both agents. The maximal tolerable dose (MTD) was defined as the dose with 0–1 DLTs. The recommended phase II dose (RPTD) would be the MTD or, absent significant toxicities, full doses of both agents.

Once the MTD/RPTD was determined, 20 additional patients were recruited at this dose level to better define the safety and clinical activity of the regimen, as well as to collect biomarker data. These subjects received single agent therapy at the RPTD, with either ABT-510 or bevacizumab monotherapy followed by combination therapy. This design allowed for the comparison of changes in biomarkers related to each drug individually and in combination.

### Patient evaluations

Baseline screening was performed within 2 weeks of the initiation of protocol therapy. Safety evaluations were performed weekly during cycle 1 and at the beginning of each cycle and as clinically indicated for all subsequent cycles. Assessments included physical examinations with vital signs and performance status as well as complete blood count with differential, liver function test and chemistry panel. UPCR, electrocardiogram (ECG), and coagulation studies were performed on the first day of each cycle. Radiographic examination and relevant blood tumor markers were assessed every other cycle. General symptom management and supportive care were provided as clinically indicated to provide optimal patient care. Adverse events were graded using the National Cancer Institute common toxicity criteria, version 3.0 [12].

Tumor response was assessed by Response Evaluation Criteria in Solid Tumors (RECIST) criteria every 8 weeks [13]. Patients could continue to receive ABT-510 and bevacizumab until disease progression, unacceptable adverse event(s), consent withdrawal, or lack of available drug supply.

### Biomarkers

Blood was collected from each patient by venipuncture into a sodium citrate vacutainer (BD Vacutainer, catalog # 369714; Beckton Dickinson, Franklin Lakes, NJ) at baseline, at the end of cycle 1 and every 2 cycles thereafter. After mixing, the tubes were centrifuged at 2500 × g for 15 minutes. The upper layer of plasma was transferred to a fresh tube and centrifuged one more time at 2500 × g for 15 minutes. This double-spun, platelet-poor plasma was aliquoted, snap frozen, and stored at −80°C until use. Plasma samples were analyzed using Searchlight platform (Aushon Biosystems, Inc., Billerica, MA) following manufacturer's protocol for the protein analytes listed in Table 2. Briefly, samples were thawed on ice, centrifuged at 20 000 × g for 5 minutes to remove any residual precipitate and appropriately diluted before placement onto SearchLight plates in duplicate. Samples and standards were incubated at room temperature for 1 hour while shaking. Plates were washed three times using a plate washer (Biotek Instruments, Inc., Model ELx405, Winooski, VT), biotinylated secondary antibody added, and incubated for an additional 30 minutes. After three more washes, streptavidin-HRP was added to the plates, incubated for 30 minutes, washed again, and substrate added. Images of the plates were taken within 10 minutes, followed by image analysis using SearchLight array analysis software.

**Table 2:** List of plasma proteins analyzed using SearchLight platform

Soluble angiogenic factors		Matrix-derived angiogenic factors	Markers of coagulation	Markers of vascular activation and inflammation	
ANG-2	PEDF	MMP2	CRP	E-cadherin	NT-proBNP
bFGF	PlGF	MMP9	D-dimer	E-selectin	MCP-1
HGF	VEGF-A	Osteopontin	PAI-1 active	Gro-α	P-selectin
IGFBP1	VEGF-C	TGFβ1	PAI-1 total	ICAM-1	SDF-1
IGFBP3	VEGF-D	TGFβ2	Tissue factor	IFNγ	TNFα
PDGF-AA	sVEGFR1	TSP1	vWF	IL-6	VCAM-1
PDGF-BB	sVEGFR2	TSP2		IL-8	

ANG-2, angiopoietin-2; bFGF, basic fibroblast growth factor; HGF, hepatocyte growth factor; IGFBP, insulin-like growth factor-binding protein; PDGF, platelet-derived growth factor; PEDF, pigment epithelium-derived factor; PlGF, placental growth factor; VEGF, vascular endothelial growth factor; sVEGFR, soluble vascular endothelial growth factor receptor; MMP, matrix metallopeptidase; TGFβ, transforming growth factor beta; TSP, thrombospondin; CRP, c-reactive protein; PAI-1, plasminogen activator inhibitor-1; vWF, von willebrand factor; Gro-α, growth regulated oncogene-alpha; ICAM-1, intercellular adhesion molecule 1; IFNγ, interferon gamma; IL, interleukin; NT-pron-BNP, N-terminal prohormone brain natriuretic peptide; MCP-1, macrophage chemoattractant protein-1; SDF-1, stromal cell-derived factor-1; TNFα, tumor necrosis factor-α; VCAM-1, vascular cell adhesion molecule 1.

### Wound angiogenesis assay

Skin biopsies were obtained using previously reported methods [14]. Briefly, before drug treatment, a 4-mm skin biopsy was performed to stimulate granulation (wound) tissue formation, and 7 days later, a 5-mm biopsy was performed in the same location to collect the resulting granulation tissue. Before each biopsy, the patient's skin was locally anesthetized with 1% lidocaine with epinephrine at a ratio of 1:100,000, mixed with sodium bicarbonate (8.4%). Topical antibiotic ointment followed by a nonocclusive bandage was applied to each wound; patients were given written and verbal wound care instruction guidelines.

Stimulus and granulation tissue biopsies in the biomarker expanded cohort were performed according to the schedule as follows: day 7 (baseline stimulus), day 1 (baseline granulation), day 8 (monotherapy stimulus), day 15 (monotherapy granulation), day 22 (combination therapy stimulus), and day 29 (combination therapy granulation). Dermal neovascularization at the wound periphery was evaluated using a digital camera with a special dermatologic adapter (Heine Dermatophote). All the images were scored using an ordinal 0–4 scoring system of wound vascularization, with each wound field divided into 12 clock hours. Each section was scored by two independent observers blinded to treatment status or image timing, and an average vascular score (AVS) was obtained for each time point [15] Granulation tissue samples (5-mm biopsies) were homogenized and assessed by Western blot using antibodies to total-VEGFR2 (Santa Cruz Biotechnology, Inc, Cat# sc-315, Santa Cruz, CA) and phospho-VEGFR2 (Cell Signaling Technology, Cat#2478, Beverly, MA). ELISA assays for TSP-1 and 2 (R&D Systems, Minneapolis, MN) were also performed on the protein lysates according to manufacturer's directions. All assays were done in duplicate.

### Statistical analysis

Descriptive statistics were used to summarize demographic and baseline disease characteristics. Biomarker assessments were all considered exploratory and therefore the sample size of 20 patients for the expanded cohort was based upon practical consideration. For each plasma-based biomarker, one-sample Wilcoxon signed-rank tests were performed to investigate whether the log fold change was different from 0 between end of cycle 1 and baseline. For the analytes at *P* < 0.05, average log fold change and 95% confidence intervals were calculated. For AVS, the median changes and their ranges were tabulated. To explore any potential correlation between changes in blood biomarkers and AVSs, Spearman's correlation coefficients were computed. SAS version 9.2 and R 2.12 (SAS Institute, Inc., Cary, NC) were used for the statistical analyses.

## Results

### General

Between September 2005 and June 2008, 37 patients were enrolled; a total of 34 patients received bevacizumab at doses ranging from 5 to 10 mg/kg IV every 14 days and ABT-510 at doses ranging 50 to 100 mg subcutaneously twice daily. Of these, 32 were evaluable for response and 33 were evaluable for toxicity. Patient characteristics are listed in Table 3. Patients were treated in 3 dose levels and in a biomarker expanded cohort at the MTD/RPTD (Table 1).

**Table 3:** Patient characteristics

Characteristic	No. of patients
Total patients	34
Gender	
Male	13
Female	21
Age (yrs) Median (range)	56 (19–74)
Karnofsky performance status	
Median (range)	90 (70–100)
Number of prior treatments (chemotherapy, radiation)	
0	5
1 or 2	9
3 or 4	15
>5	6
Primary tumor type	
Colorectal	10
Pancreatic neuroendocrine	2
Esophageal	2
Fibrous histiocytoma	2
Cholangiocarcinoma	2
Others^1^	16

1 Includes one patient each with breast, ovarian, thyroid, melanoma, hurthle cell, cervical, hepatocellular, desmoid, duodenal, adrenal corticoid, sarcoma NOS, giant cell, endometrial, osteosarcoma, clear cell carcinoma.

### Safety/Toxicity

Adverse events for all dose levels are listed in Table 4. There was one DLT at dose level 3, a treatment-related hospitalization for gastrointestinal bleed due to a gastric varix that required endoscopic intervention. Although the varix was likely pre-existing, a contribution to the bleeding event from study treatment was deemed likely. There were no DLTs at lower dose levels. The most common adverse events seen (epistaxis, injection site reaction, headache, fatigue, and anorexia) were grade 1/2; with most of these events being grade 1. In addition to the bleed due to the gastric varix, there was one grade 3 anorexia and one other GI bleed; both of these events were felt to be treatment related. No significant hematologic toxicity was observed and no patient experienced a grade 4 or 5 treatment-related adverse events.

**Table 4:** Treatment-related grade adverse events *N* = 33

	Grade 1 No.	Grade 2 No.	Grade 3 No.	Grade 4 No.
Class-related effects				
Hypertension	0	0	0	0
Proteinuria	0	0	0	0
Epistaxis	9	0	0	0
Thrombosis	0	0	0	0
Gastrointestinal				
Diarrhea	3	2	0	0
Constipation	5	0	0	0
Nausea	4	2	0	0
Vomiting	0	1	0	0
GI bleed	0	0	2	0
Other				
Anorexia	4	0	1	0
Fatigue	5	4	0	0
Headache	6	0	0	0
Injection site reaction	15	0	0	0
Rash	9	0	0	0

### Efficacy

In this population of heavily pretreated patients with solid tumors, there was one RECIST-defined partial response (PR) in a patient with thyroid carcinoma treated at the RPTD who had a 41.2% decrease in disease measurements; this patient remained on study for 20+ cycles. One patient treated at dose level 1 and 5 patients treated at the RPTD had prolonged stable disease for >1 year. This included one patient each with pancreatic adenocarcinoma (16 cycles), desmoid tumor (15 cycles), metastatic giant cell tumor (17+ cycles), endometrial adenocarcinoma (18+ cycles), cholangiocarcinoma (12 cycles), and neuroendocrine tumor of the pancreas (14+ cycles). Of note, four patients achieving prolonged disease control (denoted by “+”) stopped treatment due to lack of availability of ABT-510. Four additional patients had stable disease for at least 6 months but less than a year.

### Biomarker analysis

#### Plasma-based biomarkers

**Figure 1:**
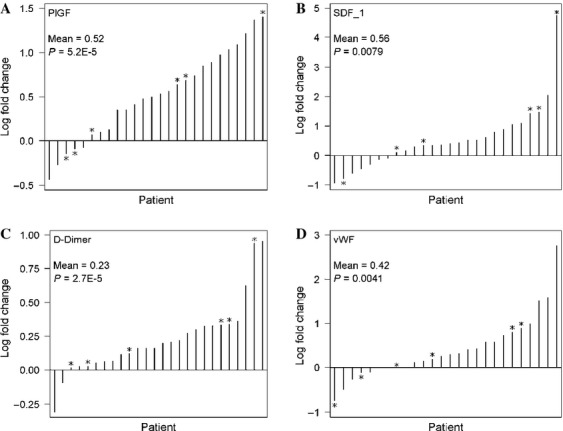
Waterfall plots of the fold change in PlGF (A), SDF-1 (B), D-dimer (C), and vWF (D) plasma levels on drug treatment in all patients. Each bar represents the log2 fold change (end of cycle 1/baseline) of the plasma analyte in a single patient, arranged in ascending order. *Patients that had PR or SD >1 year. The mean log2 ratios and *P*-value (one-sample Wilcoxon signed-rank test) are included in the inset of each graph. SDF-1, stromal-derived factor 1; PlGF, placental growth factor; vWF, von Willebrand factor; SD, standard deviation.

Levels of several categories of soluble proteins, including angiogenic factors, coagulation factors, and markers of vascular activation and inflammation, were analyzed at baseline and at the end of cycle 1. All patients were on combination therapy at the end of cycle 1. For technical reasons, samples could not be analyzed at time points when patients were on ABT-510 and bevacizumab monotherapies. Levels of placental growth factor (PlGF) and stromal-derived factor 1 (SDF-1), two markers previously described to change with anti-VEGF treatment, were significantly increased in response to combination treatment in this study (Fig. 1A and B) [16–18]. D-dimer and von Willebrand's Factor (vWF), two coagulation-related markers not previously described to change in response to either treatment, were also significantly increased after one treatment cycle (Fig. 1C and D). No consistent changes in other analytes between baseline and cycle 1 were observed. Due to the heterogeneous population and small size of this study, we did not attempt to correlate baseline analyte levels or changes in analyte levels to clinical outcome.

#### Wound angiogenesis assay

##### AVS analysis

A novel wound angiogenesis assay was incorporated into this study to determine if the effects of these antiangiogenic therapies, bevacizumab and ABT-510, could be monitored using a dermal wound assay. While not statistically significant, AVS scores were modestly decreased after combination drug treatment (data not shown). Spearman-based correlations between changes in AVS scores and changes in circulating plasma markers were performed. Changes in transforming growth factor beta1 (TGFβ1) and TGFβ2 plasma levels were found to modestly correlate to changes in AVS with correlation coefficients of ρ = −0.64 and −0.63, respectively. While unadjusted *P*-values of TGFβ1 and TGFβ2 were significant at *P* < 0.01, more stringent multiple parameter testing resulted in false discovery rate (benjamini–hochberg procedure) of 0.13 for both.

##### Tissue VEGFR2 and TSP analysis

No consistent changes in the levels of total VEGFR2 or phospho-VEGFR2 were observed in the granulation tissue across the cohorts with drug treatment, for either monotherapy or for combination treatment. Levels of TSP1 and TSP2 protein in granulation tissue also did not significantly change with either monotherapy or with combination treatment.

## Discussion

This study evaluated the tolerability, antitumor, and antiangiogenic activity of the combination of ABT-510 plus bevacizumab in patients with refractory solid tumor malignancies. Overall, the combination was very well tolerated; most toxicities were grade 1 or 2. There was one grade 3 gastrointestinal bleed related to a previously unrecognized gastric varix, which may have been precipitated by study treatment, and one grade 3 anorexia felt to be related to study treatment. Grade 1/2 irritation at the ABT-510 injection site and grade 1/2 epistaxis were occasionally seen. No other toxicities traditionally associated with antiangiogenic agents, including cardiac ischemia, hypertension, proteinuria, poor wound healing, and/or bowel perforation, were observed.

With the small number of evaluable patients in this study (34 treated in total and 28 treated at the RPTD) and tumor heterogeneity, it is difficult to make any conclusions about efficacy of this combination. There was one PR (thyroid carcinoma). While several antiangiogenic multikinase inhibitors have shown activity in thyroid cancer, most of these agents also inhibit the ret oncogene [19]. The activity of bevacizumab in thyroid cancer has not been evaluated. Stable disease lasting at least 6–12 months was noted in four patients (all at the RPTD); stable disease lasting at least 12 months was noted in six patients (1 at dose level 1 and 5 at the RPTD). Three of these patients stopped treatment due to drug supply issues not due to toxicity or disease progression. Of the patients with prolonged stable disease, several had tumor types (neuroendocrine tumor of the pancreas, endometrial carcinoma, and sarcoma) for which antiangiogenic therapies have reported activity. Interestingly, for patients with potentially sensitive tumor types, the duration of stability seen in patients on this trial compares favorably to the median progression-free survivals reported in the literature [20–22]. In addition, several patients with tumor types not known to benefit from anti-VEGF therapy (duodenal adenocarcinoma, squamous cell carcinoma of the cervix, and cholangiocarcinoma) had stable disease for at least 6 months. Whether these signs of clinical activity are related to one or both agents or to patient selection cannot be determined from the current study. The heterogeneity of histologic subtypes also makes this determination difficult.

In this study, we measured multiple plasma-based markers of angiogenesis at baseline and on-treatment; only effects on combination treatment were evaluable. Consistent with many published reports evaluating plasma biomarker responses to various VEGF inhibitors, including bevacizumab, SDF-1, and PlGF levels were increased in patients on this study [16–18].

Two coagulation factors, D-dimer and vWF, were found to be increased. D-dimer and vWF have been associated with clinical outcomes in several cancer types [23–26] and coagulation factors are known to play a key role in the generation of tumor stroma formation and in the expression and processing of VEGF and many other angiogenesis factors, including TSP, PlGF, and SDF-1 [27–30]. In a small randomized study, increases in D-dimer were reported to correlate with disease progression in patients with colorectal cancer treated with bevacizumab [23]. Bevacizumab has also rarely been associated with the development of thrombotic microangiopathies, conditions associated with changes in coagulation markers [31]. The increase in both markers in the current study were not consistent with these conditions and there was no clinical or other laboratory evidence (altered platelets, hemoglobin, PT, PTT, creatinine) for microangiopathies on this study. The biological and clinical significance of these changes are not known, particularly as this finding was noted for patients with both short and prolonged stable disease and was not associated with thrombosis.

Wound angiogenesis was only slightly affected by treatment with ABT-510 and bevacizumab as monotherapies and/or by combination therapy with both drugs, as determined by AVSs in a skin punch biopsy assay. Additionally, no consistent changes were observed in the activation state of VEGFR2 or in the levels of TSP1 or TSP2 within the granulation tissue following either ABT-510 or bevacizumab monotherapy or combination therapy. Wound healing is orchestrated by the spatial and temporal expression of proangiogenic and antiangiogenic factors; it is possible that these molecular endpoints are dynamic during treatment and our assessment at only one time point (i.e., 7 days after the wound stimulus) may have missed an effect window that might have been seen at another time point. The correlation of plasma TGFβ1 and TGFβ2 levels with a treatment effect on wound vascularization is of uncertain significance in this pilot study. However, it possible that TGFβ levels reflect states of greater or lesser host wound healing capacity and/or sensitivity to anti-VEGF or TSP mimetic therapy.

Lastly, combination antiangiogenesis therapy remains an area of active investigation. Although TSP is among the first and most promising endogenous antiangiogenesis inhibitors, developing TSP mimetics has been difficult, largely due to the large size and multifunctional roles of TSP and the lack of a well-defined receptor and signaling pathway. The TSP mimetics ABT-510 and COVX-045 are no longer in clinical development. Nevertheless, the partial response in one patient and the prolonged stable disease (≥6 months) seen in 32% of patients on this study treated at the RPTD suggest a potential role for combined anti-VEGF and TSP mimetic therapy. Despite the reported pharmacokinetic limitations of ABT-510, the clinical disease control noted above suggests that this agent may have biological activity in cancer patients. These data would support continued efforts to understand and potentially develop next generation agents in this class.

In conclusion, the combination of ABT-510 and bevacizumab was well tolerated and clinical activity was noted in a variety of tumor types. Evaluation of plasma-based biomarkers revealed up-regulation of D-dimer, vWF, SDF-1, and PlGF in response to treatment with ABT-510 plus bevacizumab. These data suggest combination antiangiogenesis therapies may be clinically useful and that several biomarker approaches can be used to describe the effect of these agents.
